# On the design principles of peptide–drug conjugates for targeted drug delivery to the malignant tumor site

**DOI:** 10.3762/bjoc.14.80

**Published:** 2018-04-26

**Authors:** Eirinaios I Vrettos, Gábor Mező, Andreas G Tzakos

**Affiliations:** 1 University of Ioannina, Department of Chemistry, Section of Organic Chemistry and Biochemistry, Ioannina, GR-45110, Greece; 2 Eötvös Loránd University, Faculty of Science, Institute of Chemistry, Pázmány P. stny. 1/A, H-1117 Budapest, Hungary; 3 MTA-ELTE Research Group of Peptide Chemistry, Hungarian Academy of Sciences, Eötvös Loránd University, Pázmány P. stny. 1/A, H-1117 Budapest, Hungary

**Keywords:** bioconjugates, cancer, drug delivery, PDC, peptide, peptide–drug conjugate, side-products in PDCs

## Abstract

Cancer is the second leading cause of death affecting nearly one in two people, and the appearance of new cases is projected to rise by >70% by 2030. To effectively combat the menace of cancer, a variety of strategies have been exploited. Among them, the development of peptide–drug conjugates (PDCs) is considered as an inextricable part of this armamentarium and is continuously explored as a viable approach to target malignant tumors. The general architecture of PDCs consists of three building blocks: the tumor-homing peptide, the cytotoxic agent and the biodegradable connecting linker. The aim of the current review is to provide a spherical perspective on the basic principles governing PDCs, as also the methodology to construct them. We aim to offer basic and integral knowledge on the rational design towards the construction of PDCs through analyzing each building block, as also to highlight the overall progress of this rapidly growing field. Therefore, we focus on several intriguing examples from the recent literature, including important PDCs that have progressed to phase III clinical trials. Last, we address possible difficulties that may emerge during the synthesis of PDCs, as also report ways to overcome them.

## Introduction

### Current cancer chemotherapy

Cancer is one of the leading causes of death globally behind the heart and circulatory disorders based on statistics of World Health Organization (WHO) [1]. Among all different types of cancer, the most fatal for males are lung and prostate cancer, while for females are breast cancer, colon & rectum cancer [1]. Notably, more than 12 million cancer cases and 7 million cancer deaths are estimated to have occurred both in males and females in 2008 worldwide [2]. These numbers have mounted up to 15 million cases and 8.8 million deaths in 2015. These statistics clearly indicate that cancer is not retreating but is creeping up, tending to become the leading cause of mortality. Thus, it can be concluded that the current therapeutic formulations utilized in oncology are not adequately effective against the complexity of cancer, mostly due to the associated collateral toxicity present in healthy tissues. It is estimated that about 30% of the clinical trials on ClinicalTrials.gov are related to cancer, while only 10% of them eventually gain market approval [3], rendering the drug development, especially in this therapeutic direction, costly and inefficient. Specifically, 12 cancer drugs were approved by the FDA in 2017 [4], comprising 26% of the total amount of approvals with respect to other therapeutic areas. These figures suggest that it is of great importance to turn the focus of the global market on targeted therapies. In 2009, the total earnings in the United States, derived from targeted cancer drugs, have reached $10.4 billion, showing an almost 2.2-fold increase since 2005 [5]. However, despite the significant attention that field has gained the past decades, it still remains unfulfilled.

**Figure 1:**
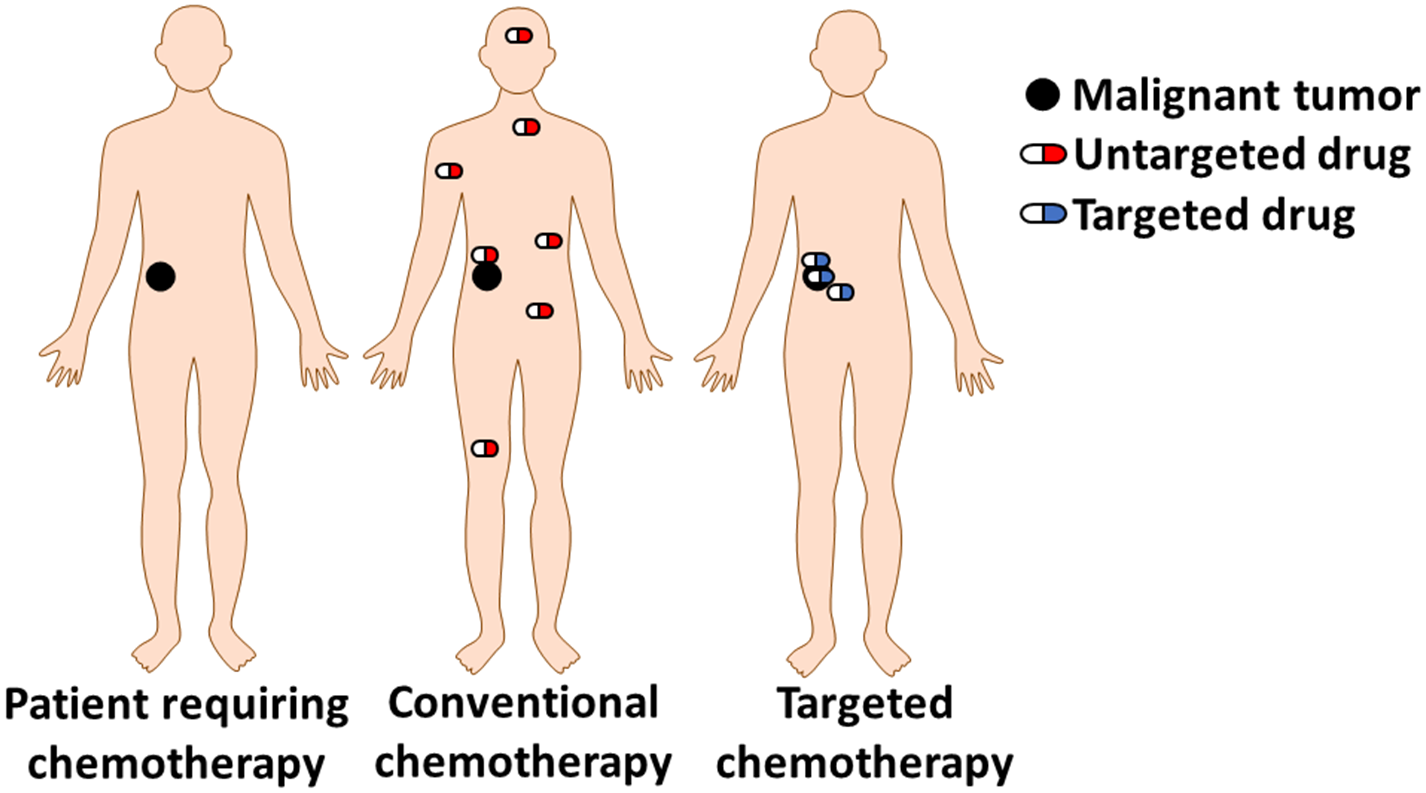
Conventional chemotherapy versus targeted chemotherapy. Black color = Solid malignant tumor; red = conventional untargeted cytotoxic agent; blue = targeted cytotoxic agent.

Current treatment processes involve a combination of surgical intervention, radiation and chemotherapy. Drugs used for this purpose are inevitably cytotoxic in order to eliminate cancer cells, but they lack selectivity that could be developed through targeting malignant cells (Figure 1). Due to the uncontrolled peripheral toxicity, anticancer drugs usually kill healthy tissues, resulting in severe effects on the patient’s health. One representative example is gemcitabine, which demonstrates higher toxicity for healthy cells, after long-term administration, with respect to cancer cells. This happens since cancer cells evolve more rapidly and develop drug resistance by diminishing expressed nucleoside receptors responsible for the cell uptake of gemcitabine [6].

Additionally, chemotherapy with anticancer agents is often hampered by their poor aqueous solubility, low oral bioavailability and metabolic instability. These drawbacks are linked to the unfavorable ADME (absorption distribution metabolism excretion) that are basically described in the following four consecutive axes: 1) Absorption is directly connected with the transportation process of the drug from the site of administration to the systemic circulation [7]. 2) Distribution refers to the delivery of the drug to the tissues which usually occurs via the bloodstream. Conventional chemotherapeutic drugs (gemcitabine, paclitaxel, doxorubicin, etc.) are not capable to be selectively delivered to the tumor sites and they end up scattered in the whole body. 3) Metabolism is a standard biological strategy for detoxification, breaking down of the administrated drugs, once inserted into the human body. The drugs get decomposed and converted to their metabolites. These metabolites can be pharmacologically inactive, e.g., gemcitabine converted to 2',2'-difluorodeoxyuridine (dFdU) [8] or possess enhanced activity with respect to the parent drug, e.g., temozolomide converted to 5-(3-methyltriazen-1-yl)imidazole-4-carboxamide (MTIC) [9]. 4) Excretion is the final step and is responsible for the removal of the parent drug and/or its metabolites from the human body. Renal excretion is the predominant route of elimination, occurring via urine.

Therefore, most conventional cytotoxic agents applied in chemotherapy lack optimum pharmacokinetic properties (ADME) and thus are not very effective to treat malignancies. Moreover, despite the intensive research to construct new cytotoxic drugs, survival rates in most cancers remain low [10] and clinical attrition rates in oncology have been devastating [11]. These data render obvious that the currently developed drugs, as also the continuous attempt to discover new ones, have not provided the expected therapeutic impact in oncology.

It is clear that we do have access to an enormous pool of unspecific cytotoxic agents that can efficiently kill cancer cells. What is currently needed is not to invest so intensively in generating more cytotoxic agents but to re-use and re-cycle available ones and tailor them to be transformed into targeted chemotherapeutics. Along these lines, drug delivery vehicles that can be tailored for different types of cancer and shape personalized therapeutics are continuously gathering attention. Such drug delivery systems are of ultimate importance to effectively surpass these hurdles and eventually improve drug potency.

### Charting the malignant tumor microenvironment

In order to selectively deliver cytotoxic drugs to malignant tumor sites, scientists can take advantage and map first the differential microenvironment between cancer and normal cells. The first one to report a fundamental difference between malignant and normal cells was Otto Heinrich Warburg in the early 1900s, who was awarded the Nobel Prize in 1931. He proposed that malignant tumor growth relies on aerobic glycolysis, in contrast to normal cells that generate energy by mitochondrial oxidative phosphorylation. The fact that cells converted pyruvate to lactate, even in the presence of oxygen, rendered his observation puzzling for scientists, who still struggle to elucidate the complete mechanism of action of diseased cells. Following the Warburg effect, ^18^F-deoxyglucose positron emission tomography (FDG–PET) imaging was developed in order to visualize the phenomenon of increased glucose uptake by cancer cells [12].

Nowadays, it has been demonstrated that malignant cells differ markedly in many metabolic aspects compared to normal cells [13], thus offering the opportunity to target them in various ways. Most cancer tissues exhibit the following characteristics that can be exploited for developing targeted cytotoxic agents:

1. Dysregulation of translation initiation factors and regulators [14].
2. Mutations in epigenetic regulatory genes [15].
3. Overexpression of surface receptors like HER2R [16], folate receptor [17], GnRH receptor [18–19] and amino acid transporters [20].
4. Overwhelming production of stimulus agents and enzymes [21]. For instance, many types of cancer show enhanced levels of reactive oxygen species (ROS) which are reactive molecules and play a crucial role in cell proliferation [22].
5. The slightly acidic pH of the tumor microenvironment [23] (Warburg effect).

These are some noteworthy differences that underlie the discrimination between cancerous and normal cells and are often exploited in order to control the site of the drug release during targeted cancer chemotherapy.

## Review

### Strategies for targeted delivery of toxic warheads to malignant tumor sites

The main challenge of the drug delivery concept is to transport sufficient amount of the cytotoxic agent to a specific location with minimum adverse side effects. To conquer this, various approaches are being exploited at the moment. These include, but are not limited to: a) utilization of drug delivery vehicles and formulates like nanoparticles [24] and calixarenes or cyclodextrins [25–26], where the cytotoxic drug is loaded and can be released at the malignant tumor site; b) installation of labile chemical groups to the tumor microenvironment (i.e., low pH) able to mask the cytotoxic drug and form a prodrug with enhanced plasma stability and/or cell permeability [27] and in the same time diminished toxicity for normal cells; c) covalent attachment of a drug on a tumor-targeting element (small molecule, peptide or antibody) able to selectively target and permeate cancer cells. The conjugation is being conducted via a rationally designed linker able to release the drug inside the cancer microenvironment [19].

**Figure 2:**
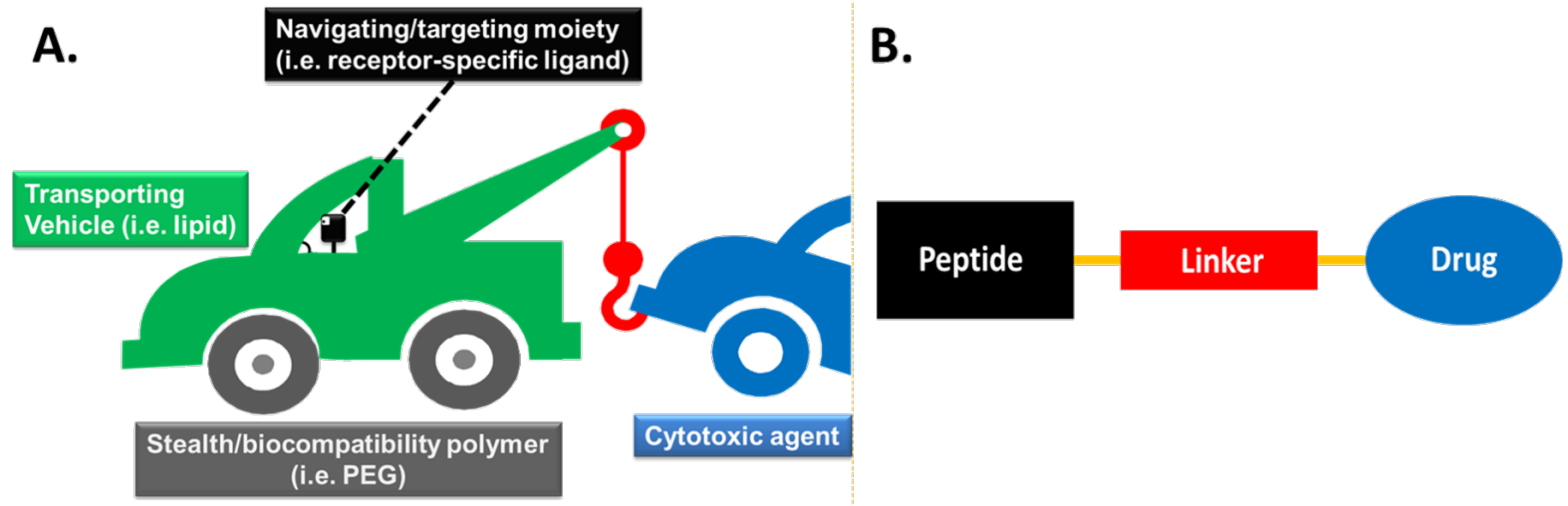
A. General structural architecture of the ideal navigated drug delivery system [31]. B. General structure of a peptide–drug conjugate (PDC).

The ideal targeting molecular device would consist of the following modules: a) the cytotoxic agent (drug), b) the transporting - drug delivery vehicle (i.e., lipid, mannan [28–30]), c) the linker tethering the transporting vehicle to the cytotoxic warhead, d) the “programmable” navigating/targeting moiety (i.e., receptor-specific ligand) and e) the “stealth” carrier (i.e., PEG) transfusing enhanced bioavailability. These modules are encoded in Figure 2A with different colors: the transporting vehicle in green color, the drug in blue color, the linker in red color, the navigating/targeting agent in black color and the “stealth” carrier in grey color [31]. The specific color coding will be followed, for simplicity purposes, in all examples of targeting devices that will be presented throughout this review.

Among the most intriguing navigating delivery systems that can combine the transporting vehicle and the navigating/targeting moiety in a single module are the tumor-homing peptides [32]. These peptides are exploited to assemble the peptide–drug conjugates (PDCs) which are considered as prodrugs, due to the covalent coupling of a peptide to a drug via specific linkers. The main building blocks of a simple PDC include a cytotoxic agent (drug), a tumor-homing peptide (navigating/targeting moiety) and a linker between them (Figure 2B).

This class of prodrugs is continuously gaining attention since peptides can be easily produced in large quantities and their purification is simple. Moreover, an array of different tumor-targeting peptides has been discovered [32] for multifarious types of cancer. This bountiful palette can permit the construction of personalized cancer therapeutics upon selecting a tumor-homing peptide that will be most appropriate for the type of cancer needed. In addition, peptide sequences can be selected according to the required physicochemical properties such as solubility, stability and overall charge or the characteristic groups necessary for the conjugation with the therapeutic payload. The overall experimental procedure to synthesize a PDC is usually rapid and facile. Notably, the overall cost to produce a PDC, where an already approved drug can be selected and re-used from a pool of available cytotoxic agents, is much lower compared to the cost of synthesizing a new cytotoxic agent, as it is based on an already applied drug with the addition of a small peptide. Nevertheless, the last years more complex bioconjugates have been synthesized to allow the simultaneous diagnosis and therapy (theranostics) of diseases.

The therapeutic efficacy of a PDC is predominantly associated with the potency of the drug and the targeting efficiency of the assembled conjugate. Thus, PDCs should possess certain features to render them appealing candidates for treatment:

1. The peptide contained in the PDC must bind selectively and with the optimal affinity to a certain receptor, present on the cell surface of the targeted tissues and not within their cytosol or nucleus (i.e., steroid receptors [33]).
2. The selected receptor should be uniquely expressed or overexpressed on cancer cells (usually 3-fold or higher in comparison with normal cells). Additionally, it should be expressed in sufficient levels to pump inside the cell efficacious doses of the drug.
3. The peptide-carrier should be constructed in such way that the conjugation with a drug or/and a fluorophore is feasible. Conjugation usually occurs on lysine, cysteine and glutamic acid [34] via orthogonal coupling or on the free N-terminus of the peptide during solid phase peptide synthesis. Though, the conjugation site should be carefully selected, since perturbations induced in the peptide structural microenvironment may result in the abolishment of its binding affinity/selectivity to the targeted receptor.
4. The linker should be carefully selected to succeed the optimal performance of the PDC. An injudicious selection may cause diminished binding affinity of the peptide to the receptor or/and reduction of the therapeutic window of the drug. Additionally, it should be enzymatically stable during the blood circulation in order to efficiently reach the malignant tumor site and release the payload in its microenvironment, reducing the off-target toxicity.
5. The cytotoxic agent should contain proper functional group that can be linked to the tumor homing peptide (i.e., gemcitabine [19]) or if it is not present it should be rationally installed taking into consideration the final derivative of the cytotoxic agent to retain the original cytotoxic activity. The sections below summarize the basic design principles of peptide–drug conjugates to selectively target the malignant cells.

### Selecting the proper tumor-targeting peptide to generate the PDCs

There is an immense variety of peptides (linear or cyclic) that have been exploited as carriers/targeting elements to successfully deliver the cytotoxic warhead to cancer cells [32]. These peptides are cell-specific and bind to certain receptors promoting their internalization. They are usually inserted into the cell via endocytosis and then they are transported to intracellular compartments with higher concentration of enzymes and lower values of pH, where they disassociate from the receptor and afterward from the anticancer agent. The most representative examples of peptides utilized for PDCs are highlighted below.

Linear peptides are included among the rich reservoir of options, finding applications in tumor targeting. They exist in different lengths, structures and with various physicochemical properties.

Attempting to ameliorate certain disadvantages of linear peptides like fast renal clearance or low binding selectivity/affinity due to the unstable structure of the linear peptides, cyclic peptides have been introduced. An immense number of cyclic peptides have been synthesized [35–37] and many of them have displayed superior affinity and selectivity for the receptor than their parent linear counterparts [38]. Cyclic peptides are usually synthesized by reacting the N-terminus with the C-terminus or by exploiting specific functional groups of certain amino acids present in the sequence. A representative example is the sulfhydryl group of cysteine-containing peptides which may cyclize through the formation of intramolecular disulfide bonds [39].

The most commonly used linear peptides and cyclic peptides that can be delivered inside cancer cells via endocytosis and one that smuggles into glioma tissues via transcytosis (angiopep-2) are presented below:

**Arginine-glycine-aspartic acid (RGD):** A widely applied peptide carrier is the tripeptide arginine-glycine-aspartic acid (RGD) motif, which was first identified by Ruoslahti and Pierschbacher in the early 1980s [40] within fibronectin that mediates cell attachment and was known to target integrin α5β1 [41]. In general, the ‘integrin’ nomenclature was first used in 1987 to describe a family of receptors, appearing as heterodimers of noncovalently associated α and β subunits, able to link the extracellular matrix (ECM) with the intracellular cytoskeleton to mediate cell adhesion, migration and proliferation [42]. The RGD motif is contained in various proteins like fibrinogen, fibronectin, prothrombin, tenascin and other glycoproteins [43] and is known to be recognized by over 10 integrins, among the over 24 known integrins [44–45], including all αv integrins, α5β1, α8β1 and αIIbβ3 [46].

The fact that carcinogenesis is highly dependent on migration, invasion and angiogenesis renders integrins important anticancer targets. Integrin αvβ3 is an important factor in tumor angiogenesis and metastasis [45], two common characteristics of cancer that discriminates it from other diseases. Notably, integrin αvβ3 (also known as the vitronectin receptor) appears to be the most important among all integrins regarding cell proliferation, invasion and angiogenesis [47]. This integrin is overexpressed on activated endothelial cells, new-born vessels and other tumor cells [48–49], but it is found to be expressed at undetectable levels in most adult epithelial cells, making it a suitable target for anti-angiogenic therapy [50]. Due to its high levels of expression in cancer cells, several peptides containing the RGD motif have been exploited for the formulation of PDCs, with the most representative example to be the peptide CDCRGDCFC [46,51–52].

**Gonadotropin-releasing hormone (GnRH):** Gonadotropin-releasing hormone (GnRH), also known as luteinizing hormone-releasing hormone (LHRH), is a hormone responsible for the secretion of two gonadotropins: follicle-stimulating hormone (FSH) and luteinizing hormone (LH) from the anterior pituitary gland. GnRH is synthesized and released from GnRH neurons within the hypothalamus and selectively binds to its receptor (GnRH-R), a seven-transmembrane G-protein-coupled receptor. The structure of the GnRH hormone (pGlu-His-Trp-Ser-Tyr-Gly-Leu-Arg-Pro-Gly-NH_2_) was first discovered in 1971 by Baba et al. [53]. Besides this form, there is GnRH-II (pGlu-His-Trp-Ser-His-Gly-Trp-Tyr-Pro-Gly-NH_2_) discovered in most vertebrates as well as in humans [54]. This peptide acts through a similar receptor (type II GnRH-R), which is expressed in different tissues, including tumor cells. Another natural isoform of GnRH is GnRH-III (pGlu-His-Trp-Ser-His-Asp-Trp-Lys-Pro-Gly-NH_2_), which has been isolated from sea lamprey. GnRH-III binds to GnRH-R overexpressed on the cancer cell surface, resulting in an antiproliferative effect but seems to be less potent than the rest GnRH analogs regarding stimulating gonadotropin release at the pituitary level [55].

GnRH peptide analogs constitute an emerging class of tumor homing peptides for malignant tissues expressing the GnRH-R. Their development is based on the fact that specific human cancer cells (mostly ovarian, prostate, lung and breast) uniquely express or overexpress GnRH-R with respect to normal tissues [55–57]. Therefore, covalent attachment of a cytotoxic agent to these peptides provides the possibility to produce potent tumor-targeting PDCs. Various amino acid alterations have been performed with respect to the native hormone [58], while the most frequently used GnRH analog is D-Lys^6^-GnRH-I, which is known to bind selectively to GnRH-R. The substitution of Gly^6^ of the native hormone with D-Lys^6^ provided an analog with higher binding affinity, stabilized β-bend and resistance to proteolytic cleavage. Moreover, the side chain of lysine contains a free amine group (εNH_2_) allowing orthogonal coupling with a cytotoxic warhead [19]. A considerable number of PDCs based on GnRH [59–63] exist and our group has exploited this peptide to construct two PDCs [18–19].

**Somatostatin (SST):** Somatostatin is a neuropeptide produced by neuroendocrine, inflammatory and immune cells and has an important role in various physiological functions acting as a classical endocrine hormone, a paracrine regulator or a neurotransmitter [64]. Somatostatin appears in two distinct active forms: somatostatin-14 (SST-14) and somatostatin-28 (SST-28). Both SST-14 and SST-28 exhibit biological activity through high-affinity membrane receptors (somatostatin receptor 1–5; SSTR1–5), that are widely distributed throughout the human body in various tissues like the nervous, pituitary, kidney, lung and immune cells [65–66].

SSTRs are overexpressed in various neuroendocrine malignant tumors (NETs) including pancreatic, pituitary, prostate, lung carcinoids, osteosarcoma etc. and other non-NETs including breast, colorectal, ovarian, cervical etc. [67]. Therefore, these receptors can be targeted for selective delivery of efficient concentrations of cytotoxic warheads to the tumor sites. However, native somatostatin gets rapidly hydrolyzed due to enzymatic degradation and therefore, more stable and potent analogs have been developed. These analogs were synthesized by replacing L-amino acids with their D-isomers and reducing the length by keeping only the peptide epitope responsible for the biological activity. The most widely known analogs of somatostatin are cyclic peptides named octreotide (*d*-Phe-*c*[Cys-Phe-*d*-Trp-Lys-Thr-Cys]-Thr-ol), lanreotide (*d*-2Nal-*c*[Cys-Tyr-*d*-Trp-Lys-Val-Cys]-Thr-NH_2_) and vapreotide (*d*-Phe-*c*[Cys-Tyr-*d*-Trp-Lys-Val-Cys]-Trp-NH_2_), which bind mainly to the subtype 2 receptor (SSTR2) that is known to be the most frequently overexpressed SSTR [68]. There are several examples of PDCs consisting of the aforementioned somatostatin targeting peptides [67,69–70], as also other somatostatin peptide analogs, e.g., pentetreotide (DTPA-*d*-Phe-*c*[Cys-Phe-*d*-Trp-Lys-Thr-Cys]-Thr-ol) [71].

**Epidermal growth factor (EGF):** Epidermal growth factor receptor (EGFR) is a transmembrane protein belonging to the ErbB family of receptor tyrosine kinases which consists of 4 structurally-related members: EGFR/HER1 (ErbB-1), HER2/neu (ErbB-2), HER3 (ErbB-3) and HER4 (ErbB-4). Cohen and Rita Levi-Montalcini shared the Nobel Prize in Medicine in 1986 for discovering growth factors. EGFR is upregulated in a wide pool of cancer tissues and is able to enter cells usually via clathrin-mediated endocytosis [72]. Many peptides have been discovered to bind the EGFR with high affinity and selectivity through screening phage display libraries and have been used like a viable approach for targeted drug delivery: YHWYGYTPQNVI [73], CMYIEALDKYAC [74], LTVSPWY [75], YWPSVTL [76].

**Angiopep-2:** A peptide that has recently attracted attention is a 19-mer peptide named angiopep-2 (TFFYGGSRGKRNNFKTEEY), due to its ability to cross the blood-brain barrier (BBB). The BBB is formed by the endothelial cells of the brain, restricting and controlling the exchange of molecules between the central nervous system and the rest body. Angiopep-2 is able to cross the BBB via receptor-mediated transcytosis after binding to the low-density lipoprotein receptor-related protein-1 (LRP-1), which is overexpressed in brain cells [77]. Moreover, the two lysines available in its sequence render angiopep-2 an appealing PDC candidate, with the aim to smuggle therapeutic payloads to brain malignancies [78–79].

**Figure 3:**
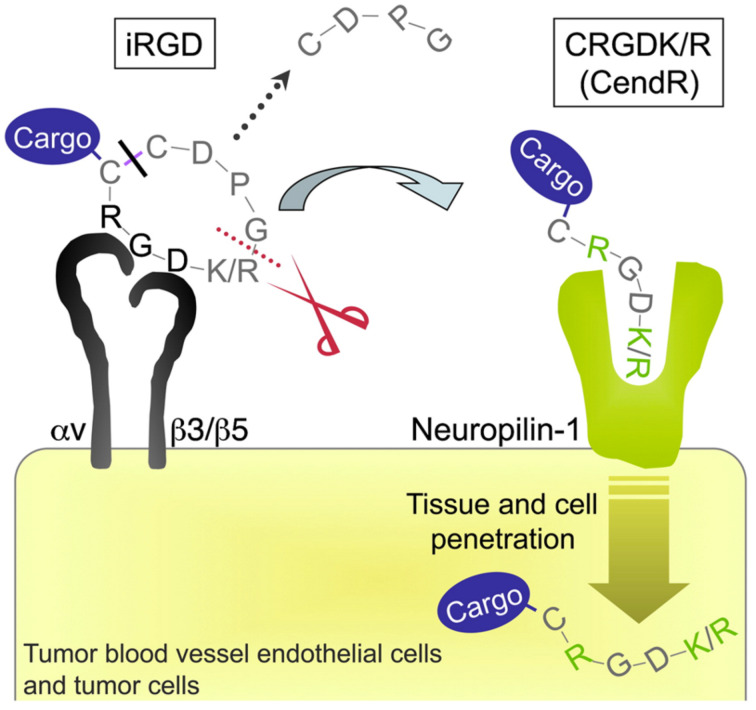
Binding and penetration mechanism of iRGD. The iRGD peptide is accumulated on the surface of αv integrin-expressing endothelial and other cells in malignancies. The RGD motif is responsible for binding to integrins. Afterward, the peptide is cleaved by cell surface-associated protease(s) to eventually expose the cryptic CendR element, RXXK/R, at the C-terminus (red dotted line). The CendR element then interferes with the binding to neuropilin-1, resulting in tissue and cell penetration. The tumor-penetrating peptide can be used to decorate a cargo (a simple chemical moiety or a nanoparticle), but only in the case that the cargo is attached to the N-terminus of the iRGD peptide as the disulfide bond is cleaved before the peptide is internalized (black line). The figure was adopted from reference [81] (© 2009 Elsevier Ltd.).

Cyclic peptide variants have been developed for the RGD peptide motif, reported above. The most commonly used cyclic peptide is iRGD (CRGDKGPDC), a 9-amino acid cyclic peptide, with tumor tissue penetration activity [80]. iRGD initially binds to αVβ3 and αVβ5 integrins that are overexpressed in tumor endothelial cells. Afterward, a proteolytical cleavage takes place to reveal a cryptic RXXK/R motif located at the C-terminus (CendR motif, C-End Rule), which then binds to neuropilin-1 (NRP-1), activating an endocytic transport pathway responsible for the enhanced transport of anti-cancer drugs into tumors (Figure 3) [80].

In Table 1 are reported the most common peptides (linear and cyclic) utilized in PDCs.

**Table 1:** The most common peptides (linear and cyclic) utilized for the formulation of PDCs used in cancer. Letters with bold color stand for D-amino acids.

peptide name	peptide sequence	targeted receptor	reference

RGD	R-G-D	integrin αvβ3	[37,46,51–52]
iRGD	CRGDK/RGPD/EC	integrin αvβ3/αvβ5	[81]
octreotide	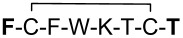	SSTR2/5	[69]
D-Lys^6^-LHRH	Glp-H-W-S-Y-**K**-L-R-P-G	LHRH-R	[18–19,61]
angiopep-2	T-F-F-Y-G-G-S-R-G-K-R-N-N-F-K-T-E-E-Y	LRP-1	[78–79]
GE11	Y-H-W-Y-G-Y-T-P-Q-N-V-I	ErbB1 (EGFR)	[73]

### Selecting the proper cytotoxic agent to generate the PDCs

**Figure 4:**
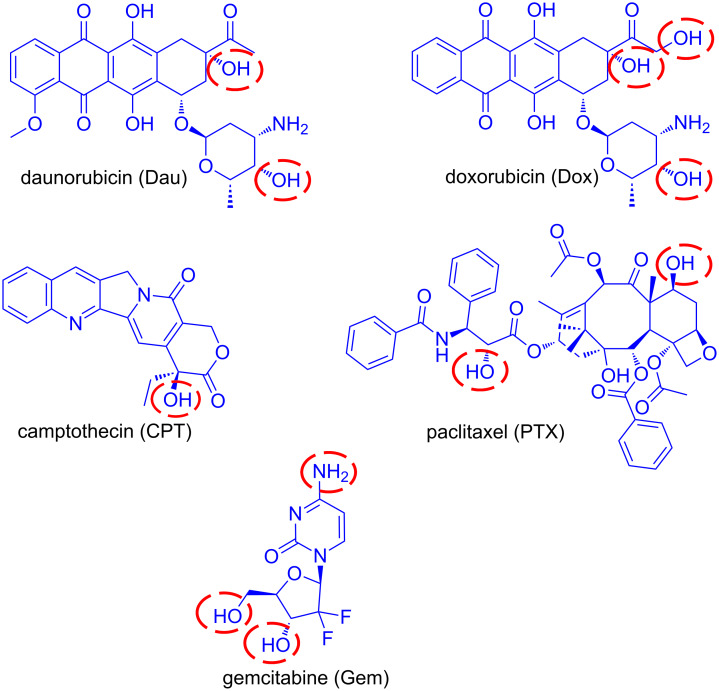
Representative examples of anticancer drugs utilized for the construction of PDCs. The most usual conjugation sites are marked with red cycles.

According to the National Cancer Institute (cancer.gov), there are more than 250 FDA-approved anticancer drugs utilized to treat malignancies at the moment. Among this large pool of cytotoxic drugs, an array of them has been utilized as toxic warheads in PDCs and five representative examples are gemcitabine, doxorubicin, daunorubicin, paclitaxel and camptothecin (Figure 4). The main drawback of these original anticancer agents is their uncontrolled toxicity which results in severe side effects. Without the addition of a targeting moiety, they bear low capacity to discriminate cancerous from normal cells. Moreover, the addition of a peptide as a targeting vehicle can enhance the pharmacokinetic and therapeutic window of the parent cytotoxic agent. Since different drugs may employ a different mechanistic approach to kill cells, the appropriate drug is selected according to features characterizing the targeted cancerous cells. For instance, daunorubicin and doxorubicin possess similar mechanisms of action [82], whereas gemcitabine [83], camptothecin [84] and paclitaxel [85] function through different mechanisms.

The selected drug must comply with certain design principles in order to serve as an appealing candidate for PDCs. The selected drug must be amenable to the linker chemistry. It must bear an intrinsic functional group for direct conjugation with the peptide/linker (Figure 4) or a functional group able to be derivatized for further conjugation (i.e., click chemistry [86]). In the latter case, the site of derivatization has to be carefully selected so that the biological activity of the drug and the release of the active drug will not be perturbed. In case that the drug binds through recognition of a specific receptor, in silico approaches have to be recruited in order to rationally select the location of the drug that will be chemically modified [18].

Furthermore, it must be sufficiently cytotoxic versus the selected malignant tumor cells in order to eliminate them and consequently promote tumor regression. The selected drug should ideally possess low-nanomolar IC_50_ values for the targeted malignant tumor. A legitimate strategy to overcome a low drug potency problem is by increasing the drug loading of the peptide-carrier. For example, in the PDC ANG1005, 3 drug molecules (paclitaxel) were loaded on a single angiopep-2 peptide which has completed phase II clinical trials [87]. Nevertheless, the concept of higher drug loading is hard to be implemented, in contrast with single drug loading that is usually preferred, mostly due to poor physicochemical properties.

Below we analyze a set of drugs that have been tailored and incorporated in PDCs.

**Gemcitabine (Gem):** Gemcitabine (dFdC) is a nucleoside analog of deoxycytidine in which the hydrogen atoms on the 2' carbon are replaced by fluorine. It is sold under the brand name Gemzar by Eli Lilly and Company and has been FDA approved for the treatment of various cancers including breast, ovarian, non-small cell lung and pancreatic cancer. The main drawbacks for its use are the high and non-selective toxicity to normal cells, the deactivation through deamination in its inactive metabolite dFdU, the acquired multidrug resistance (MDR) and its high hydrophilicity deterring its prolonged drug release from various vehicles [88], which therefore reduces the effective concentration of gemcitabine. It enters cells through nucleoside transporters hENTs (human equilibrative nucleoside transporters) and hCNTs (human concentrative nucleoside transporters) and mostly through hENT1 (human equilibrative nucleoside transporter 1) [89–90]. After internalization, gemcitabine is sequentially mono-, di- and tri-phosphorylated by phosphorylating kinases. Gemcitabine diphosphate (dFdCDP) and gemcitabine triphosphate (dFdCTP) are the active metabolites which inhibit processes required for DNA synthesis [91]. The incorporation of dFdCTP into DNA during polymerization, which causes DNA polymerases unable to proceed, is the major mechanism by which gemcitabine causes cell death (masked termination) [83]. Regarding the possible functional sites in gemcitabine that can be used for the construction of PDCs are its primary and secondary alcohols as also the amine (Figure 4).

**Paclitaxel (PTX):** Paclitaxel (PTX) is a member of the taxane family and one of the most common anticancer agents used against a wide variety of tumors. It is sold under the brand name Taxol by Bristol-Myers Squibb Company and is FDA approved for the treatment of breast cancer, ovarian cancer, non-small cell lung cancer and AIDS-related Kaposi's sarcoma. The main disadvantages in the utilization of paclitaxel are its high hydrophobicity, requiring suitable vehicles to effectively deliver it to tumor tissues, and the development of multidrug resistance due to the P-glycoprotein-mediated efflux [85,92]. Paclitaxel stabilizes microtubules by binding specifically to the beta-tubulin subunit, promoting mitotic halt and consequently cell death [93]. The difference with other known drugs that act on microtubules (vinca alkaloids) is that paclitaxel does not induce the disassembly of microtubules but boosts the polymerization of tubulin [94]. Sites available in PTX for the formation of PDCs are highlighted in Figure 4.

**Anthracyclines:** Anthracyclines are among the main anti-cancer drugs that are applied in combinations with other chemotherapeutic agents. They are utilized against a variety of cancers including leukemias, lymphomas, breast, ovarian, bladder and lung. Daunorubicin (Dau) was the first anthracycline discovered that was extracted from *Streptomyces peucetius*, a species of actinobacteria, at the beginning of the 1960s. Shortly after, the isolation of doxorubicin (Dox) from a mutated *Streptomyces* strain was accomplished. Anthracyclines are consisted of a tetracyclin aglycon part and a daunosamine sugar moiety. The difference between Dau and Dox is a hydroxy group substituted at the C-14 carbon atom on Dox providing an extra conjugation site for ester linkage (Figure 6). The mechanism of action of anthracyclines is based on their intercalation to DNA inhibiting the macromolecular biosynthesis. Furthermore, they stabilize the topoisomerase II DNA complex preventing the transcription. They may also increase quinone type free radical production, however, this plays a role rather in their cytotoxic side effects. Daunorubicin is mainly used in the treatment of leukemia [95] while doxorubicin in the cure of other types of cancers (breast cancer, bladder cancer, Kaposi's sarcoma) in combination with other anti-cancer agents.

**Camptothecin (CPT):** Camptothecin is a cytotoxic alkaloid collected from extraction of the bark and stem of the Chinese tree ‘Camptotheca acuminata’. It was first isolated and characterized in 1966 by Wall et al. [96–97]. The main mechanism of action involves binding to the reversible complex of topoisomerase I (topo I) and the 3′-phosphate group of the DNA backbone through hydrogen bonding, resulting in accumulation of a persistent ternary complex (the cleavable complex). This stabilized complex prevents the re-ligation step of DNA, catalyzed by topo I, resulting in DNA damage and therefore cell death (apoptosis). CPT is predominantly cytotoxic during the S phase replication of DNA because of the collision of the replication fork with the cleavable complex, converting the single-strand breaks into double-strand breaks and eventually causing cell death [98]. CPT can be conjugated to targeting elements to enhance its efficacy via its primary alcohol marked in Figure 4. Although CPT showed remarkable results during its phase I clinical trials against a variety of solid tumors, its low water-solubility and stability led to the formulation of various new analogs with the same mechanism of action. The two most progressed analogs of CPT are topotecan and irinotecan. Topotecan (hycamtin) has been approved by the FDA for the treatment of ovarian and cervical cancer, as also small cell lung carcinoma. Irinotecan (camptosar) has been approved by the FDA for the treatment of metastatic carcinoma of the colon or rectum, alone or in combination with fluorouracil (5-FU). Camptothecin has been utilized as an anticancer agent in various PDC formulations, such as conjugation with the targeting peptides D-Lys^6^-LHRH [99], somatostatin [100] and c(RGDyK) [101].

### Linker design for PDCs: Principles and representative examples

Another crucial aspect that should be considered during the design of a PDC is the linker tethering the peptide and the drug. The linker has to be carefully shaped so as not to perturb the binding affinity of the peptide to its receptor and the drug efficacy. An inappropriate linker may impede the release of the drug from the PDC and therefore diminish its overall therapeutic potency. Linkers utilized in PDCs exist in different categories and vary on their length, stability, release mechanism, functional groups, hydrophilicity/hydrophobicity etc.

This linker can be designed to bear an enzyme-hydrolyzable unit (EHU) like a carboxylic ester or an amide bond, cleaved by esterases and amidases, respectively. The most commonly utilized linkers that bear a carboxylic ester bond, as the enzyme-hydrolyzable unit, are succinyl (derived from succinic acid) and glutaryl (derived from glutaric acid). Concerning the utilization of amide bond in the linker as the unit tethering the drug and the peptide, it can be tailored to be cleaved based on the targeted tissue and/or type of cancer where a specific protease is statistically upregulated (i.e., cathepsin B upregulated in various malignancies including lung, brain, prostate and breast [102]). Also, during the design of the PDC specific attention has to be given on the selection of the bonds that will be used in the linker. Specifically, in several currently available PDCs, at least two different bonds are used: one to connect the linker to the peptide and the other to connect the drug to the linker. Such cases have to consider, during the design process, the microenvironment that the assembled PDC is to be located, since different enzymes and/or the tumor microenvironment might trigger the improper release of the drug from the PDC, i.e., to end up with the drug-carrying part of the linker.

Another class of linkers is the stimuli-responsive/degradable linkers, designed to achieve an efficient release of the drug from the bioconjugate in the tumor microenvironment. Such linkers are rationally designed to be cleaved when they sense specific stimuli in the environment of cancerous cells (slightly acidic pH, enhanced levels of reducing agents and/or enzymes) or external stimuli (ultrasound, temperature, irradiation). Specifically, there are certain bonds like imine, oxime, hydrazone, orthoester, acetal, vinyl ether and polyketal [103] that are known to undergo hydrolysis at acidic pH, while being extremely stable during blood circulation. Therefore, acid-labile bonds could be hydrolyzed in the slightly acidic microenvironment and/or in the acidic cellular compartments of cancer cells and consequently release the active drug. Additionally, disulfide linkers are often adopted in PDCs, since they are cleaved by reducing agents like cysteine and glutathione, present in high concentrations in malignant cells.

Linkers bearing enzyme-hydrolyzable units (EHU) responsive to proteases are degradable peptide linkers that have attracted significant interest due to the specificity of certain enzymes and there has been a dramatic escalation over in the past years. The most representative examples in this field are the MMP-2/9 (matrix metalloproteinases) and cathepsin B peptide substrates. MMP-2/9 and cathepsin B are proteolytic enzymes present at elevated levels in cancer cells known to participate in human tumor invasion and metastasis. Cathepsin B is able to recognize specific peptide sequences like Val-Cit (valine-citrulline) [104] and GFLG [105]. On the other hand, GPLGIAGQ [106], PLGLAG [107] and GPVGLIGK [108] are some common peptide substrates for MMP-2 and MMP-9.

**Figure 5:**
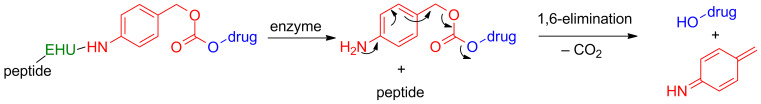
Illustration of the drug release mechanism from the self-immolative spacer PABC conjugated to a tumor homing peptide via an enzyme-hydrolyzable unit. Red color = the self-immolative spacer PABC; blue color = drug; green color = enzyme-hydrolyzable unit (EHU); black color the tumor-homing peptide.

Another rapidly emerging category in PDC linkers that has gained much attention in the last years are the self-immolative or self-destructive spacers/linkers [109–110]. This type of linkers/spacers offers the capability to release the active drug after simultaneous cascade reactions, as shown in Figure 5. *Para*-amino benzyl alcohol (PABC; colored in red) is a representative example that can be connected in the amino group via an amide bond to an enzyme-hydrolyzable unit (EHU; colored in green) and to a tumor-targeting element (i.e. tumor homing peptide; colored in black). The alcohol group at the opposite site can be connected via a carbonate ester/carbamate bond to the cytotoxic agent (colored in blue). The EHU is designed so as to be a substrate for proteases overexpressed in the targeted tumor microenvironment (i.e cathepsin B). Once EHU will be recognized by these enzymes it is cleaved off resulting in the consequent release of the active drug through rapid cascade reactions (Figure 5).

The most representative examples of various types of linkers are summarized in Table 2.

**Table 2:** Representative examples of biodegradable/responsive linkers utilized for the formulation of PDCs in cancer.

linker	drug release mechanism	reference

succinyl	action of esterases/amidases	[19]
glutaryl	action of esterases/amidases	[67]
PABC	1,6-elimination	[109–110]
oxime bond	hydrolysis in acidic pH	[111]
peptide GFLG	action of cathepsin B	[105]
peptide PLGLAG	action of MMP-2/9	[107]

### Representative examples of PDCs targeting cancer cells

Integrating the basic design principles in PDCs pinpointed above, a list of representative developed examples is analyzed below, so as to provide a spherical perspective regarding peptide–drug conjugation chemistry.

Currently, there are two PDCs that have been developed utilizing peptides as tumor targeting elements that selectively bind to specific receptors and small molecules as anticancer agents that have reached phase III clinical trials (Table 3) for the treatment of various types of cancer. ClinicalTrials.gov have also announced the initiation of a clinical trial based on various PDCs consisted of two novel peptides selected after phage display that target murine A20 leukemic cells (ClinicalTrials.gov Identifier: NCT02828774). These clinical trials will focus on chronic lymphocytic leukemia (CLL).

**Table 3:** Peptide–drug conjugates consisting of peptides and small molecules that have been used in clinical trials.

peptide	cytotoxic agent	linker	drug release mechanism	name	target	CCT^a^	reference

D-Lys^6^-LHRH	Dox (SM)^b^	glutaryl	esterases/amidases	AEZS-108	LHRH-R	phase III	[112]
angiopep-2	PTX (SM)	succinyl	esterases/amidases	ANG1005	LRP-1	phase II	[79]

^a^CCT= current clinical trials; ^b^SM= small molecule.

Except these two PDCs, there are other types of PDCs that do not consist of peptides as targeting moieties and small molecules as drugs and have reached even up to phase III clinical trials. These PDCs are summarized in Table 4.

**Table 4:** Various other types of peptide–drug conjugates in clinical trials.

peptide	cytotoxic agent	linker	drug release mechanism	target	name	CCT^a^	reference

CNGRCG	hTNFα (Protein)	–	amidases	CD13 receptor	NGR015	phase III	[113]
polyglutamic acid	PTX (SM)^b^	–	esterases	–	CT2103	phase III	[114]
LHRH	CLIP71^c^ (lytic peptide)	–	amidases	LHRH-R	EP-100	phase I	[115]
DRDDS (spacer)	DAVBLH^d^ (SM)^b^	2-mercapto- ethanol	glutathione	folate receptor	EC145	phase III	[116]
D-γ-E-γ-E-γ-E-E (masking moiety)	12ADT^e^-Asp	–	PSMA	PSMA	G-202	phase II	[117]

^a^CCT = current clinical trials; ^b^SM = small molecule; ^c^CLIP71 = KFAKFAKKFAKFAKKFAK; ^d^DAVBLH = desacetyl vinblastine hydrazide; ^e^12ADT = 8-*O*-(12-aminododecanoyl)-8-*O*-debutanoyl thapsigargin.

Notably, there is only one PDC in the market designated ^111^In-DTPA-*d*-Phe^1^-octreotide, which is utilized for diagnostic radiology in somatostatin receptor-positive tumors [118]. It constitutes a complex of ^111^Indium bound to diethylenetriaminopentaacetic acid (DTPA), which is conjugated to the targeting somatostatin peptide [D-Phe^1^]-octreotide. Recently, another similar analog, designated ^111^In-DTPA-*d*-Phe^-1^-Asp^0^-*d*-Phe^1^-octreotide, has been evaluated and presented enhanced tumor accumulation in pancreatic tumor cells and simultaneously lower renal radioactivity [119].

Herein, we will analyze in depth the two PDCs in clinical trials consisted of peptides and small molecules (Table 3), as also various other similar PDC formulations existed in the current literature that have been evaluated in preclinical models.

**Figure 6:**
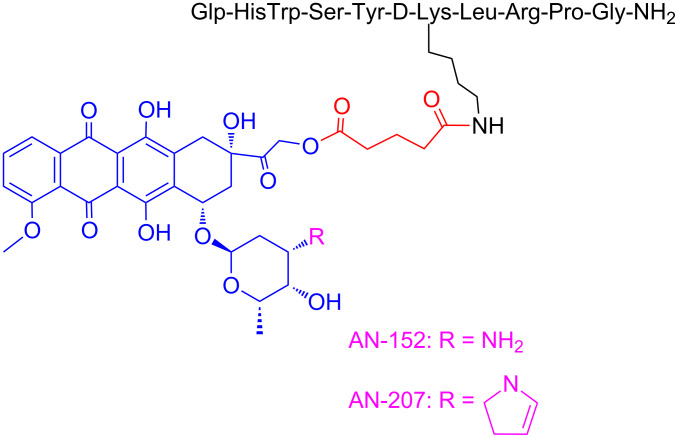
Structures of the PDCs named AN-152 and AN-207.

First, two widely-known peptide–drug conjugates named AN-152 (AEZS-108) and AN-207 will be analyzed. These conjugates contain the luteinizing hormone-releasing hormone (LHRH) as the peptide-targeting module and doxorubicin (DOX) or its daunosamine-modified derivative 2-pyrrolino-DOX as the cytotoxic agent, respectively (Figure 6). Specifically, Andrew V. Schally and his group first synthesized the corresponding analogs [120] where they covalently coupled the two drugs to the epsilon-amino group of the D-Lys side chain of the peptide D-Lys^6^-LHRH.

Notably, both conjugates fully preserved the cytotoxic activity of the parent drugs, DOX or 2-pyrrolino-DOX, respectively, in vitro and also retained the high binding affinity of their peptide carrier to receptors for LHRH on rat pituitary [120]. The two conjugates were subjected to stability tests and they showed slow drug release in human serum in contrast with nude mice that carboxylesterase enzymes are about 10 times higher [121]. Consequently, the two analogs were heavily evaluated in in vivo models in nude mice bearing various types of cancer. Mice bearing OV-1063 (LHRH receptor positive) or UCI-107 (LHRH receptor negative) human epithelial ovarian cancers were treated with AN-152 or DOX with systematic intraperitoneal administration. The growth of UCI-107 cells was not inhibited by AN-152 but systemic administration of AN-152 in OV-1063 cells proved that AN-152 is less toxic but inhibits tumor growth better than equimolar doses of DOX [122]. These results were confirmed in nude mice bearing other ovarian human cancers (ES-2), where AN-207 caused up to 59.5% inhibition in tumor growth [123]. Also, AN-207 and AN-152 were tested in female BDF mice bearing estrogen independent MXT mouse mammary cancers, presenting stronger tumor inhibitory effects than their respective cytotoxic radicals up to 93%, while equimolar quantities of their respective radicals were more toxic [124]. Moreover, PDC AN-207 was significantly more potent, regarding the growth inhibition of hormone-dependent Dunning R-3327-H prostate cancers in rats, reaching up to 50% of the initial tumor volume in comparison with 2-pyrrolino-DOX. Shortly afterward, they tested the two conjugates in membranes of human breast cancer cells: MCF-7 hormone-dependent and MDA-MB-231 hormone- independent [125]. They proved that the specific analogs retained the high binding affinity of the D-Lys^6^-LHRH carrier to the relevant receptors. Both conjugates displayed IC_50_ values in the low nanomolar concentration range for MCF-7 (13.7 ± 1.09 nM for AN-152 and 6.08 ± 0.5 nM for AN-207) and MDA-MB-231 (5.60 ± 1.24 nM for AN-152 and 1.89 ± 0.4 nM for AN-207) cells. AN-152 was tested regarding the inhibition of tumor growth of subcutaneously (sc) implanted androgen-dependent LNCaP and MDA-PCa-2b and androgen-independent C4-2 prostate cancers, xenografted into nude mice. The results demonstrated the stronger inhibition of AN-152 on the tumor with respect to the free DOX [126]. Similarly, in vivo experiments were conducted regarding AN-207 in nude mice bearing xenografts of MDA-PCa-2b prostate cancer cells, showing identical results like AN-152 [127]. Gründker et. al. evaluated the antitumor effects of AN-152 in vivo in human LHRH-R-positive HEC-1B endometrial and NIH:OVCAR-3 ovarian cancers, and in the LHRH-R-negative SK-OV-3 ovarian cancer cell line via intravenous injections [128]. The tumor volumes of HEC-1B and NIH:OVCAR-3 cancers were reduced significantly even after 1 week of treatment with AN-152 while presenting no toxic side effects. Treatment with DOX arrested tumor growth but did not reduce tumor volume. The growth of SK-OV-3 cancers was not affected by AN-152. Based on the presented results, it can be concluded that these two analogs possess higher antitumor activity but less toxicity with respect to the parent drugs DOX and 2-pyrrolino-DOX and can be used versus a wide variety of ovarian, prostate, endometrial and breast tumors.

Notably, after the extensive evaluation of analog AN-152 in preclinical models, starting from 2006 it has been tested in phase I and phase II studies (AN-152 was renamed to AEZS-108 for the clinical trials) of LHRH-R positive recurrent endometrial and ovarian cancers. The phase I/II study in castration-resistant prostate cancer (CRPC) and chemotherapy refractory bladder cancer also showed promising results. Due to the promising results from phase II trials in endometrial cancer, a multinational phase III clinical study is underway [112].

It is important to note that despite the fact that analog AN-207 presented a better biological profile, evident in all the preclinical models, its further development fell short due to chemical and plasma instability.

According to ClinicalTrials.gov, during phase I analog AEZS-108 was tested in 17 women with epithelial cancer of the ovary, endometrium or breast and for which standard treatment could not be used or was no longer effective. The results showed promising tolerance from the patients with fewer side effects than the commonly applied drugs. Moreover, AEZS-108 was evaluated in phase I clinical trial on patients with castration- and taxane-resistant prostate cancer and the results proved that AEZS-108 possesses a sufficient safety profile and efficacy. It succeeded in lowering the PSA levels in some patients with prostate cancer and it became evident that the internalization of AEZS-108 in prostate cancer circulating tumor cells (CTCs) may be a viable pharmacodynamic marker [129].

These promising results led to phase II clinical trials to patients with castration- and taxane-resistant prostate cancer and their disease showed progression after taxane-based chemotherapy. AEZS-108 showed significant activity in these patients who were pretreated with taxanes and maintained an acceptable safety profile [130].

Last, phase II clinical trials were conducted in collaboration with the German Gynecological Oncology Group (AGO) and 3 other centers from Bulgaria on 43 women. The patients had platinum-resistant advanced ovarian cancer, FIGO (Fédération Internationale de Gynécologie et d'Obstétrique) III or IV or recurrent endometrial cancer (EC) and LHRH receptor-positive tumor status. The treatment with AEZS-108 had significant activity and low toxicity in these women [131–132].

**Figure 7:**
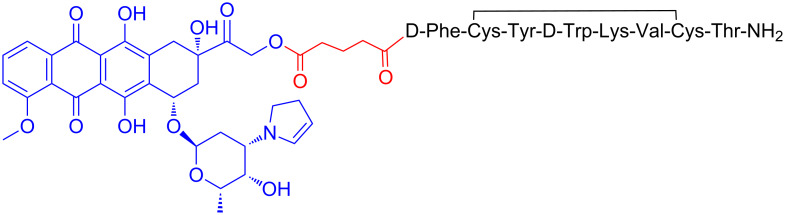
Structure of the PDC named AN-238.

Based on the fact that the previously described analog AN-207 showed superior in vitro and in vivo results compared to AN-152 but lacked stability, Andrew V. Schally and his group turned their focus on its building block 2-pyrrolino-DOX and tried to construct new PDCs using other peptides. Therefore, they synthesized a new analog, designated AN-238, consisting of the octapeptide RC-121 linked through the α-amino group of its N-terminal D-Phe moiety and a glutaric acid spacer to the 14-OH group of 2-pyrrolino-DOX (Figure 7). The octapeptide RC-121 was utilized due to its high binding affinity to the somatostatin receptor (SST-R) [133].

The anti-cancer activity was first evaluated in various rat/human cancer lines xenografted into nude mice with breast human tumors (MDA-MB-238, MCF-7, MX-1) and prostate rat/human tumors (Dunning AT-1, PC-3). All cell lines showed a great response to the treatment with AN-238 with high inhibition of the tumor, while 5 of 10 mice with MX-1 tumor were totally cured [61]. The cytotoxic profile of this analog was similarly evaluated in additional cancer cell lines xenografted into nude mice including prostate, renal, mammary, ovarian, gastric, colorectal and pancreatic [134]. Various types of renal, colorectal, pancreatic and gastric cancers showed a major response to the treatment with more than 70% inhibition while all the other types showed a good response to the treatment with an average of 60% inhibition. AN-238 was also evaluated in U87-MG brain cancer cells with good response, inducing 82% growth inhibition of subcutaneous tumors [134]. Therefore, AN-238 has been proved to be a promising candidate for a large number of tumors, being able to suppress the growth of these tumors and their metastases. Last, Engel et. al. showed that AN-238 inhibits tumor growth in human experimental endometrial carcinomas which express SST receptors, regardless of the expression levels of multidrug resistance protein MDR-1 [135]. The analog AN-238 is still pending for clinical trials.

**Figure 8:**
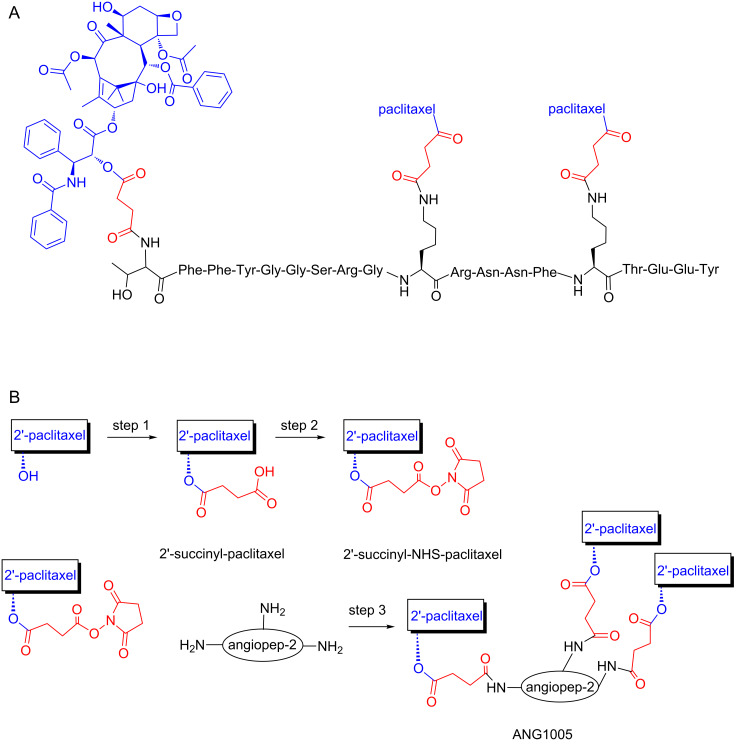
Chemical structure and synthetic scheme for the PDC ANG1005. (A) ANG1005 is composed of three molecules of paclitaxel linked by a cleavable succinyl ester linkage to the angiopep-2 peptide. (B) Schematic representation of ANG1005 synthesis steps. Paclitaxel was first reacted with succinic anhydride and then activated with *N*-hydroxysuccinimide to form 2′-succinyl-NHS-paclitaxel in two steps. In the conjugation step (step 3), amines of the angiopep-2 peptide react with 2′-succinyl-NHS-paclitaxel. The scheme was modified according to *Br. J. Pharmacol. ***2008,*** 155,* 185–197 [136].

An interesting example of a PDC able to cross the blood-brain barrier (BBB), is ANG1005 [136], composed of three molecules of paclitaxel linked by a cleavable succinyl ester linkage to the angiopep-2 peptide (Figure 8).

BBB is formed by the brain capillary endothelium with very low permeability as it excludes about 100% of the large molecules and about 98% of the small molecules attempting to pass to the brain [137]. Being mandatory to surpass the BBB in order to deliver pharmaceuticals to the brain, scientists have struggled to discover either novel small molecules able to cross it through various mechanisms [138] or novel techniques able to disrupt its dense structure like ultrasound-mediated drug delivery [139–140]. The design principles on the synthesis of the specific conjugate, ANG1005, were the following: the peptide angiopep-2 is able to cross the BBB via receptor-mediated transcytosis after binding to LRP-1 and consequently it is often used as drug delivery vehicle, while paclitaxel bears cytotoxicity against glioblastoma. It has been shown that the brain uptake of ANG1005 was 4.5-fold higher compared to paclitaxel and the cytotoxicity remained higher in all cancer cell lines tested (glioblastoma U87 MG, U118, U251; lung carcinoma A549, NCI-H460, Calu-3; ovarian carcinoma SK-OV-3). It has been also proved that human tumor xenografts were inhibited more with ANG1005 than paclitaxel. Finally, mice with intracerebral implantation of U87 MG glioblastoma cells or NCI-H460 lung carcinoma cells exhibited increased survival rates after ANG1005 administration.

Because of these promising results, ANG1005 progressed to phase I clinical trials in 2007 in 63 patients with recurrent or progressive malignant glioma. It was found that ANG1005 delivers paclitaxel across the BBB and achieves therapeutic concentrations in the tumor site. It became evident that this PDC possessed similar toxicity to paclitaxel as also enhanced activity in recurrent glioma [141]. Phase II clinical trials were then initiated on patients with recurrent high-grade glioma and on breast cancer patients with recurrent brain metastases. The results have not been published yet but it has already been stated that very promising results were collected and phase III clinical trials will start shortly. Based on the overall progress of ANG1005, other similar molecules have been synthesized and studied in preclinical models [142].

**Figure 9:**
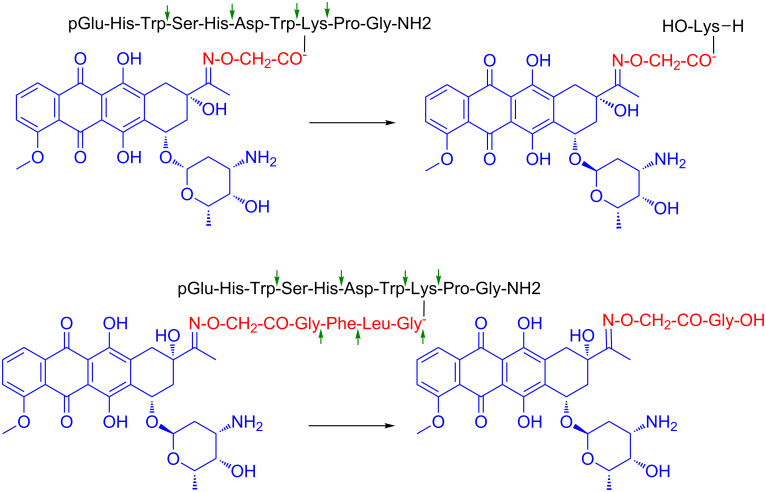
Structure of oxime linked Dau–GnRH-III conjugate with or without cathepsin B labile spacer and their metabolite released in lysosomal homogenate [144].

The group of Prof. G. Mező has achieved a great progression in the field of PDCs the last years working mostly on GnRH-III (Glp-His-Trp-Ser-His-Asp-Trp-Lys-Pro-Gly-NH_2_), which was exploited as a tumor homing device for drug targeting 10 years ago [111]. The aim of this was to apply a peptide hormone with lower endocrine effect than GnRH-I that might be useful especially for hormone-independent tumors like colon cancer [143]. In addition, GnRH-III has Lys at position 8 of the sequence providing a conjugation site without inducing perturbation in the receptor recognition. In the first conjugates, daunorubicin (Dau) was attached to the lysine side chain via oxime linkage through an aminooxyacetyl (Aoa) moiety (Glp-His-Trp-Ser-His-Asp-Trp-Lys(Dau=Aoa)-Pro-Gly-NH_2_). The oxime bond, developed between the aminooxyacetyl function and the carbonyl group of C-13 on Dau is stable under physiological conditions and prevents the early drug release in contrast to the ester bond (Figure 9).

Thus, no free drug release can be detected from such type of conjugates before reaching their targets. However, oxime bond is also stable in lysosomes where the conjugates decomposed after receptor-mediated endocytosis. Among the fragments arose during lysosomal degradation, H-Lys(Dau=Aoa)-OH was observed as the smallest Dau containing metabolite [144]. Therefore, the DNA binding propensity of this metabolite was also examined and it was found that although it is efficient it presented lower binding capacity with respect to the parent drug.

The in vitro antitumor activity of the above-mentioned conjugate was studied on MCF-7 human breast and HT-29 human colon adenocarcinoma cells [144]. The IC_50_ values showed two orders of magnitude lower effect compared to the free Dau. Thus, a systematic comparative study of various anthracycline-GnRH conjugates was conducted in order to conduct their complete evaluation as potential targeted cancer chemotherapeutics. The influence of different: (i) anthracycline drugs, (ii) linkers among the tumor-homing peptide moiety and the drug, and (iii) tumor-homing peptides (e.g., GnRH-III and D-Lys^6^-GnRH-I) was examined regarding their in vitro cellular uptake, drug release and cytostatic effect [145]. Doxorubicin (Dox) was coupled to both GnRH-III and D-Lys^6^-GnRH-I through a glutaric acid linker via ester bond. AN-152, the GnRH-I based PDC (see above), served as a control. No significant differences in cellular uptake and cytostatic effect were observed between the two PDCs. Recently, it was also indicated that the cellular uptake of carboxyfluorescein-labeled GnRH-I, GnRH-II and GnRH-III conjugates might be influenced not only by the targeting moiety, but also by the type of cancer cells [146]. However, no significant differences could be observed regarding the cellular uptake of the three GnRH conjugates by MCF-7 and HT-29 cells. It is worth mentioning that the highest water solubility was detected for the GnRH-III conjugate. The ester bond can be cleaved by esterases not only in cancer cells, but also in human plasma during blood circulation. The early drug release in the bloodstream may cause unwanted side effects. Furthermore, O–N acyl shift was detected both during the synthesis and the storage of ester-linked doxorubicin conjugate, resulting to an inactive compound where the tumor-homing peptide acylated the amine of the daunosamine sugar moiety. This was found through the mass spectrometric (MS) fragmentation profile of the PDC [146].

In a different PDC, Dau was linked to GnRH-III through a hydrazone bond or by incorporation of a self-immolative spacer [145]. The hydrazone linkage was formed similarly to the oxime bond on C-13 atom of Dau but it allows the effective drug release under slightly acidic conditions in lysosomes. The *p*-aminobenzylalcohol-based self-immolative spacer, combined with the dipeptide Lys-Phe (cathepsin-B lysosomal enzyme cleavable spacer), was connected to the amino functional group of daunosamine moiety. The last construct also provided the free drug release. Both conjugates illustrated similar cytostatic effects and cellular uptake as the conjugates with ester bonds. All these conjugates showed IC_50_ values in the range of 0.2–0.5 μM on MCF-7 cells while 1–3 μM on HT-29 cells. The free Dau or Dox had higher in vitro cytostatic effect than the conjugates, especially on HT-29 cells. Nevertheless, the synthesis of these conjugates was not so efficient and their chemical, biological and long-term shelf-stability of these PDCs were not so sufficient for drug development.

In another construct, daunorubicin and doxorubicin were attached to the ε-amino group of Lys of GnRH-III through oxime linkage [111,145]. The conjugation of the drug and the aminooxyacetylated tumor homing peptide was almost quantitative under slightly acidic conditions. Interestingly, the conjugate with Dox illustrated much lower antitumor effect than the Dau conjugate. The oxime-linked Dau-GnRH-III conjugate (non-cleavable linker) had one order of magnitude lower antitumor activity than the conjugates with the cleavable linkers. The cellular uptake of the oxime-linked conjugates was lower, too, but this effect might come from the different fluorescent properties of the free Dau and the peptide/metabolite-linked Dau. Because of the high synthetic yield and stability of oxime-linked conjugates, it can be suggested that such conjugates might be good candidates for the development of targeted tumor therapeutics. Therefore, efforts were made to develop further conjugates with higher antitumor activity.

To achieve higher antitumor activity, the sequence of the peptide GnRH-III was modified. Previous studies indicated that only a few changes are acceptable without significant loss of the anti-proliferative effect of the hormone peptide. Interestingly, Ser at position 4 could be replaced by Lys or acetylated Lys [147]. It is worth mentioning that the Ser in GnRH agonist and antagonist analogs are rarely modified [148]. The incorporation of Lys or Lys(Ac) in position 4 of GnRH-III increased the antitumor activity of the conjugate GnRH-III(Dau=Aoa). However, in the case of [^4^Lys]-GnRH-III(Dau=Aoa) enzyme stability of the conjugate was decreased while [^4^Lys(Ac)]-GnRH-III(Dau=Aoa) showed higher stability [149]. When the acetyl group was exchanged to other short-chain fatty acids (SCFAs) the enzyme stability was enhanced by the length of hydrocarbon chain of SCFAs [150]. According to the cellular uptake and cytostatic *in vitro* studies, the optimal compound was the butyric acid containing [^4^Lys(Bu)]-GnRH-III(Dau=Aoa) conjugate that almost reached the in vitro biological effects of the conjugates with a cleavable linker. This conjugate showed significant tumor growth inhibition in vivo, not only on subcutaneous implanted but also on orthotopically developed HT-29 colon cancer-bearing mice [151]. The PDC in the applied dose (15 mg Dau content/kg body weight) showed similar or higher antitumor activity than the free Dau at a maximal tolerated dose (MTD) without significant toxic side effects on organs. In contrast to the conjugate, Dau presented toxicity on the liver causing worse condition and higher mortality during the treatment.

**Figure 10:**
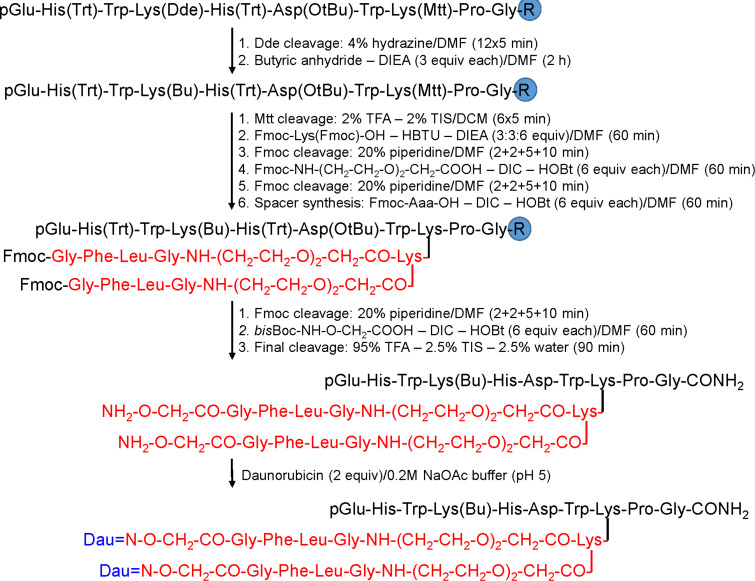
Synthesis of the most effective GnRH-III–Dau conjugate with two drug molecules [153].

In addition, the incorporation of Lys at position 4 provided a new conjugation site. Therefore, Dau or methotrexate (MTX) were attached to the ε-amino group of ^4^Lys resulting in conjugates with two identical ([^4^Lys(Dau=Aoa), ^8^Lys(Dau=Aoa)]-GnRH-III), or different drug molecules ([^4^Lys(MTX), ^8^Lys(Dau=Aoa)]-GnRH-III) [152–153]. Some improvement in the cytostatic effect could be detected compared with the conjugates containing only one drug molecule, but they were not better than the conjugate with butyric acid. This observation led to retain the Lys(Bu) at position 4 and the two Dau molecules were attached to the amino groups of an additional Lys through the enzyme labile GFLG spacer coupled to ^8^Lys (Figure 10).

The resulted PDC presented a reduced aqueous solubility, thus an oligoethylene glycol linker was inserted between the spacer and the tumor-homing peptide [154]. This PDC showed the best in vitro cytostatic effects among the oxime-linked Dau-containing conjugates, but the improvement of the synthetic process to lead to higher amounts of this PDC is required to proceed for in vivo studies. Thus, it can be concluded that oxime linked Dau–homing peptide conjugates could be good candidates for targeted tumor therapy.

**Figure 11:**
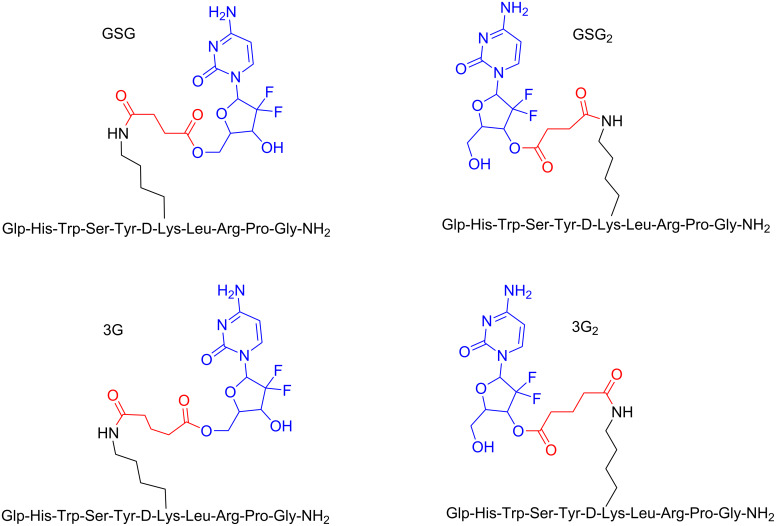
Structures of the four different PDCs of D-Lys^6^-GnRH-I and gemcitabine (GSG, GSG_2_, 3G, 3G_2_) [19].

Furthermore, the tumor homing peptide D-Lys^6^-GnRH-I has been exploited by our group to selectively deliver the anticancer agent gemcitabine to the tumor site. We, therefore, designed and synthesized four different bioconjugates consisting of D-Lys^6^-GnRH-I and the anticancer agent gemcitabine (named GSG, GSG_2_, 3G, 3G_2_) through different conjugation sites (the primary and secondary alcohol groups of gemcitabine) and using linkers of different lengths (succinyl and glutaryl) as shown in Figure 11.

In order to evaluate whether the tethering of the cytotoxic agent to the D-Lys^6^-GnRH-I peptide induces any perturbation on the local microenvironment of the peptide that is responsible for receptor recognition, we utilized ^1^H ^1^H 2D-TOCSY NMR [19]. Upon superimposing the relevant spectra of the different PDCs on the relevant spectrum of the native hormone we found that these PDCs didn’t alter the microenvironment of D-Lys^6^-GnRH-I allowing to suggest that they will not influence the binding affinity of the targeting peptide unit of these PDC to the GnRH-R. This was further validated since the new conjugates were found to possess higher binding affinity with respect to the parent peptide, with IC50 ranging even up to 1.9 nM for the conjugate 3G. The conjugates were evaluated regarding their antiproliferative effect on prostate cancer cells (DU145 and PC-3) and the PDC GSG showed IC50 values similar to gemcitabine GSG that possessed the highest antiproliferative effect was utilized for further pharmacokinetic studies in mice. These pinpointed that GSG is able to release a high amount of gemcitabine (averaging 500 ng/mL) for a period of over ≈250 min, while administered free gemcitabine was consumed in less than 100 min. At the same time, the levels of the inactive metabolite of gemcitabine (dFdU) were maintained at very low levels for the GSG conjugate in contrast to the direct administration of free gemcitabine. Finally, when injected into mice with xenografted tumors, GSG inhibited the tumor growth more effectively than gemcitabine when using equimolar quantities. Therefore, GSG could pave the way for the construction of other similar bioconjugates in order to effectively enhance the concentration of the cytotoxic drug in the tumor cells and inhibit their uncontrolled growth.

OuWe have also designed and synthesized a PDC containing the cytotoxic agent sunitinib and the D-Lys^6^-GnRH peptide-targeting-unit tethered through a succinyl linker [7]. Sunitinib is a small orally administrated drug that inhibits the phosphorylation of several receptor tyrosine kinases (RTKs). It was approved by the FDA in 2006 for the treatment of renal cell carcinoma (RCC) and imatinib-resistant gastrointestinal stromal tumor (GIST). Though, sunitinib has proved to cause severe side effects like cardiac and coronary microvascular dysfunction [155]. Therefore, these data rendered sunitinib as an appealing candidate for targeted therapy using a PDC.

**Figure 12:**
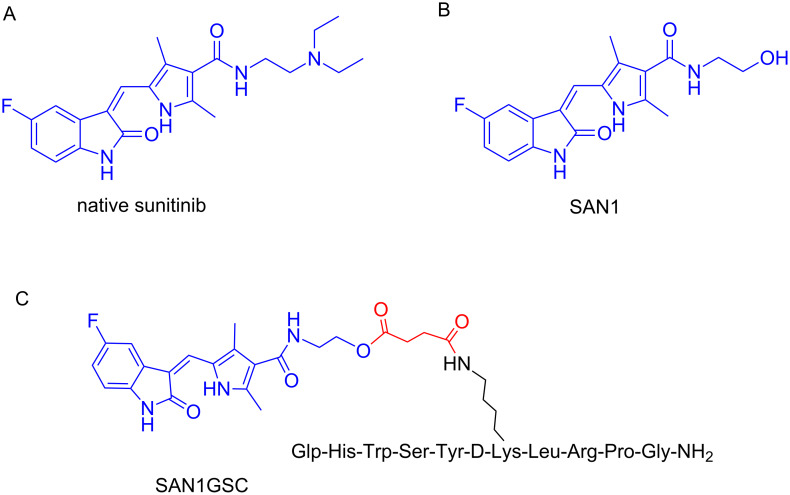
Structures of (A) native sunitinib; (B) SAN1 analog of sunitinib and (C) assembled PDC named SAN1GSC [18].

Native sunitinib (Figure 12A) does lack functional groups that could be exploited for conjugation to the peptide-targeting unit, thus, a novel analog had to be constructed (SAN1, Figure 12B). This was constructed based on in silico studies and modifying properly the drug scaffold so as not to perturb the drug binding to the targeted receptors. In silico, in vitro and pharmacokinetic evaluation of the synthesized SAN1 were conducted and compared with native sunitinib. The results indicated that SAN1 exhibited similar properties and thus could serve as an alternative to the parent drug for the formulation of the final PDC. Additionally, SAN1 was further explored in in vivo models: mice xenografted with a castration-resistant CaP (CRPC) cell line were subjected to treatment based on SAN1 and sunitinib. Mice were dosed daily via intraperitoneal injection and the results unveiled the potency of SAN1, which showed to inhibit the tumor growth in a similar way like native sunitinib. In the installed hydroxy group in the core of SAN1, a succinyl linker was conjugated that was then connected to the free amine group of Lys^8^ of D-Lys^6^-GnRH to form the PDC named SAN1GSC (Figure 12C).

SAN1GSC was evaluated in vitro and then in vivo, in mice xenografted with the CRPC model, showing similar bioactivity as SAN1. The most promising results arose from the measured concentration of SAN1 in the blood circulation and in the tumor site. The levels of free SAN1 released from SAN1GSC were 4 times higher inside the malignant cells with respect to SAN1 levels from the unconjugated SAN1. It is worth mentioning that in the frame of our construct, cardiotoxic and hematotoxic effects in treated mice were minimal and elevations of blood pressure that contribute to cardiac dysfunction were absent [18].

### Problems and solutions during synthesis of PDCs

Although the synthesis of PDCs is usually a rapid and facile procedure, various synthetic problems may arise. The most common ones appear during peptide synthesis and might refer to low aqueous solubility and/or difficulty to synthesize. Insolubility issues can be overcome by altering the C-/N-terminus and/or substituting specific residues. Difficulties in the synthesis can be handled by decreasing the number of hydrophobic residues and/or shortening the sequence. Similar synthetic problems have been encountered during peptide synthesis the last decades and have been fully addressed.

**Figure 13:**
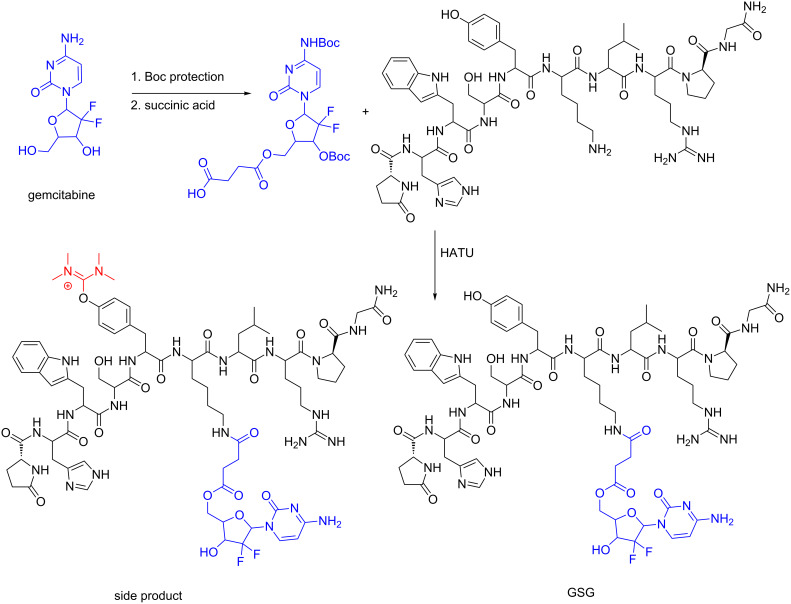
Synthetic scheme for the formation of GSG and the unexpected side product [156].

During the conjugation of 5’-*O*-gemcitabine hemisuccinate to the D-Lys^6^-GnRH peptide towards the synthesis of the PDC GSG (presented in Figure 10) we recently unveiled the formation of a previously unnoticed side product in addition to the desired product [156]. Specifically, we found that when guanidinium salts are utilized in peptide coupling conditions, a uronium derivative can be installed on specific amino acid scaffolds, beside to the formation of the expected amide bonds. This side product was persistent even after HPLC purification and was also apparent in the recorded mass spectrum of GSG as a second peak, besides the expected product, bearing the mass of the expected PDC plus 100 amu, leading to the reduction of the overall yield below 10%. Specifically, the guanidinium/uronium coupling reagent (HATU) was utilized for the formation of the amide bond between D-Lys^6^ of the peptide carrier and the carboxylic acid of the succinate linker connected to gemcitabine to synthesize the PDC GSG (Figure 13). We hypothesized that the side product was originated from the coupling reagent (HATU) and after conducting template reactions with every amino acid present in the sequence of D-Lys^6^-GnRH with Fmoc-Ser(*t*-Bu)-OH in the presence of HATU or HBTU and DIPEA, it became evident that the aminium moiety of HATU/HBTU could be installed either on the amino (–NH_2_) group of Lys or on the phenol (–OH) group of Tyr [156].

**Figure 14:**
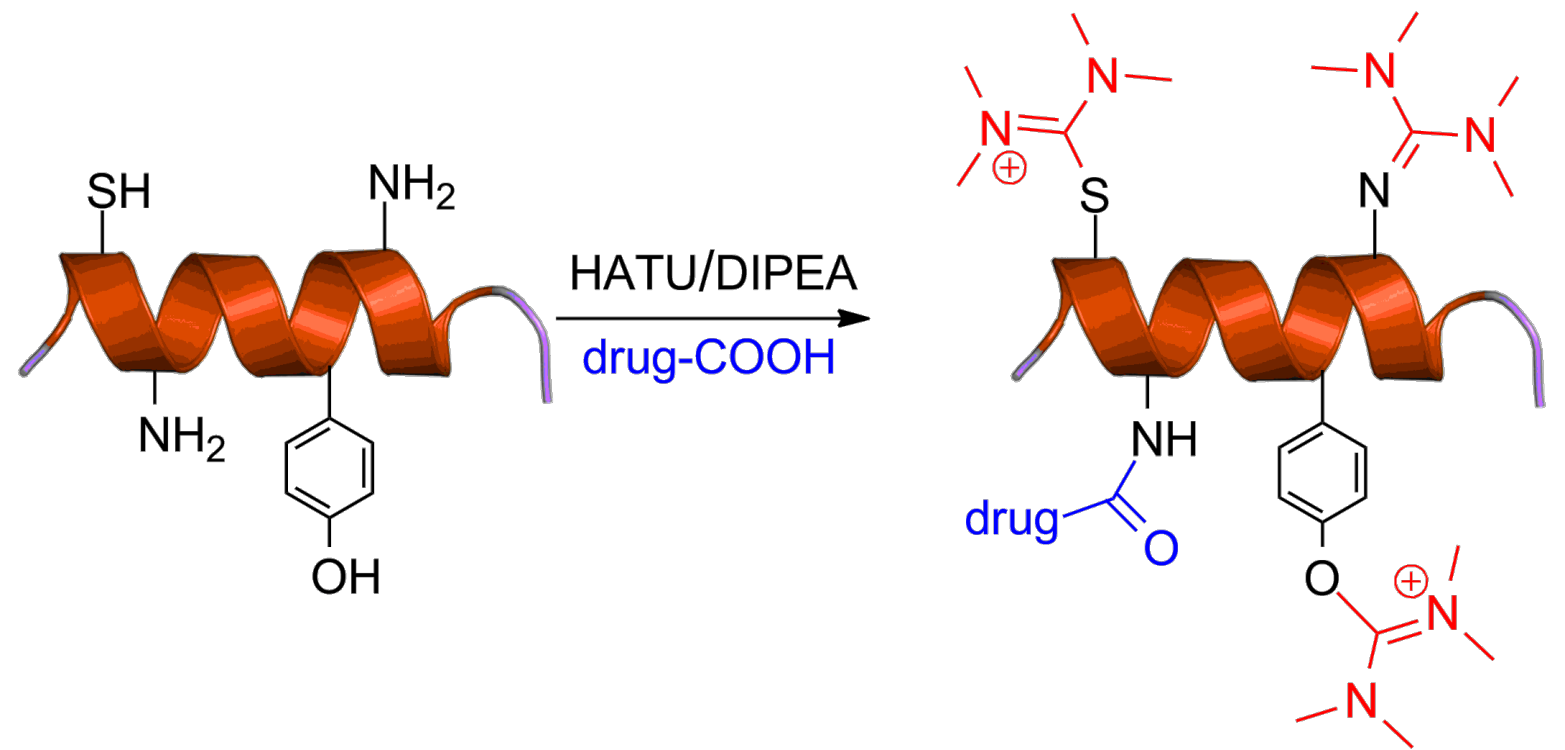
Illustration of uncharted guanidinium peptide coupling reagent side reactions during PDCs synthesis [156].

Our findings were further verified by reacting other tumor-homing peptides like D-Lys^6^-GnRH and Fmoc-HER2-BP1 (LTVSPWY, a heptapeptide known for its activity against erbB2) with HATU/DIPEA and characterizing the final products by ESIMS and ^1^H NMR spectroscopy where the same aminium side product was also recorded. Interestingly, when the dipeptide Fmoc-Cys-Tyr-NH_2_ was reacted with HATU, we found that the side product could be installed both on the phenol group of Tyr as also on the sulfhydryl group of Cys. Therefore, we tested these reaction conditions on the peptide C1B5_141–151_ subdomain peptide (RCVRSVPSLCG) of protein kinase C (PKC) γ isozymes, which possess 2 cysteines (but no tyrosine or lysine or free N-terminus amine) and bears anticancer properties [157]. Again, a side product with two aminium moieties on the two cysteines was formed and characterized with ESIMS. This observation was also applied to a simple phenol where again a side product was recorded pinpointing the broad impact of our findings beyond traditional peptide chemistry. We thus revealed the formation of a previously unnoticed side product during the synthesis of PDCs, derived from guanidinium/uronium peptide coupling reagents that occurs on phenols, primary amines and sulfhydryl groups (Figure 14).

**Figure 15:**
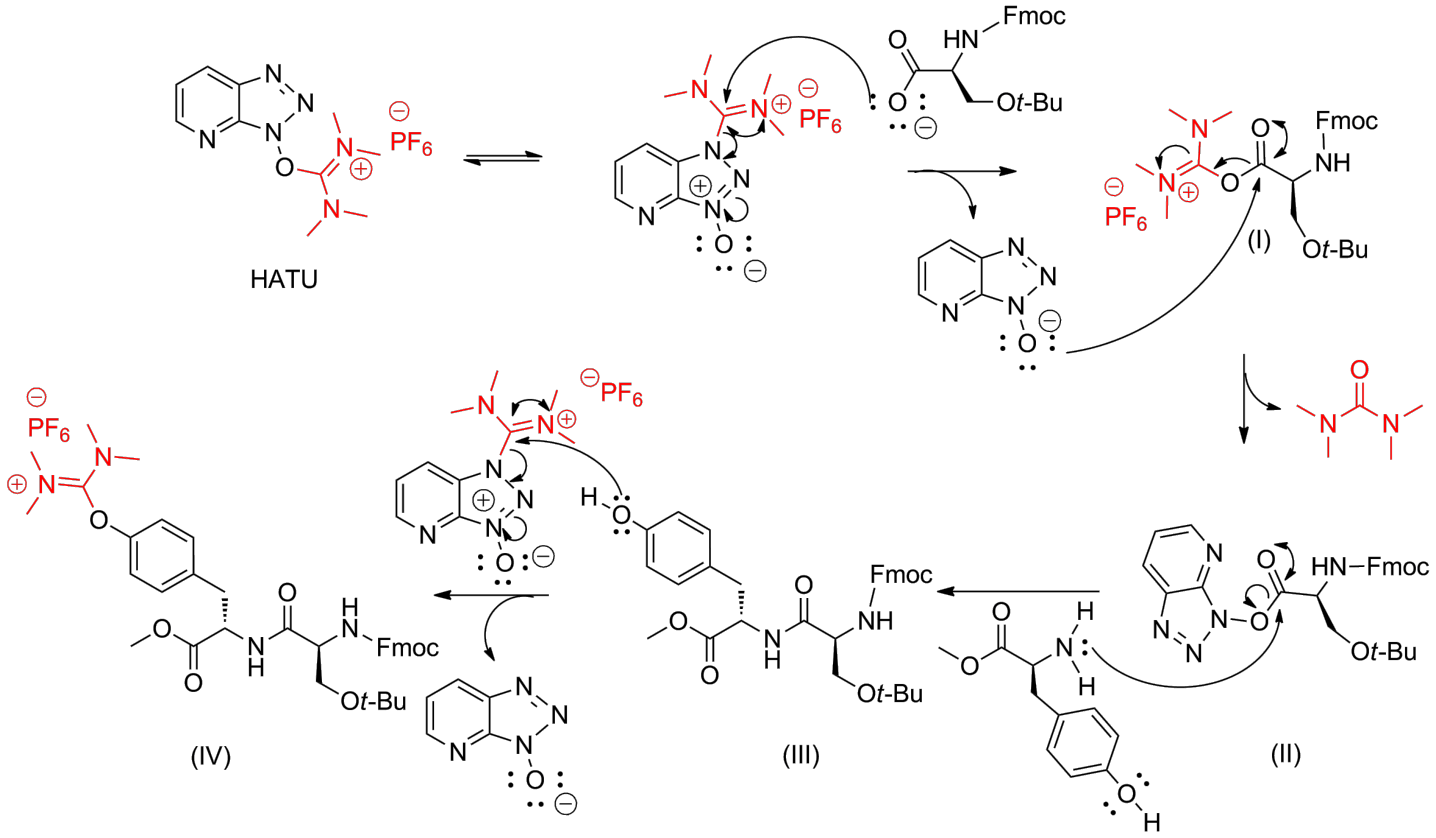
Putative mechanism for the formation of the uronium side product [156].

Along these lines, we suggested a mechanism that this side-product formation is taking place, directly after the formation of the amide bond that occurs from structure (II) to structure (III), as shown in Figure 15.

We discovered that the side product, which is difficult to be separated from the expected PDC and therefore results in reduced synthetic yield, could be avoided by using 1 equiv of HATU/HBTU, instead of the classical and established protocols using 1.5 equiv [156]. Taking into account that uronium/guanidinium coupling reagents are among the most expensive ones, using the specified conditions (equimolar quantity) may also reduce the total cost of the synthesis.

## Conclusion

Currently used chemotherapeutics are in their majority highly toxic, causing severe side effects. Thus, with the aim to enhance their narrow therapeutic index, a wide variety of strategies have been explored. Selective drug delivery via special carriers represents a viable approach to deal with tumors with higher efficacy while using lower doses of the anticancer agent. Specifically, peptide–drug conjugates (PDCs) operate as potent drug delivery carriers and thus have attracted considerable attention over the last decades. The simplicity, versatility and the relatively low cost for the construction of PDCs have rendered them appealing candidates. In the present review, basic and integral knowledge has been accumulated towards the PDCs design through examining every module required to assemble the fully decorated PDC: the peptide, the cytotoxic agent and the linker. We highlighted the overall progress of this field through selective analysis of noteworthy examples in the literature, as also possible synthetic problems that may arise and their solutions. Based on the fact that several PDCs have been selected for clinical trials and presented tumor inhibition with minimum side effects, this field needs to be further refined and explored. Through this review, we made efforts to provide an influential impetus for the construction of new peptide–drug conjugates, which could eventually transform undesired toxic drugs to highly potent formulations for the effective treatment of cancer.
